# A cost and performance comparison of Public Private Partnership and public hospitals in Spain

**DOI:** 10.1186/s13561-016-0095-5

**Published:** 2016-05-14

**Authors:** Maria Caballer-Tarazona, David Vivas-Consuelo

**Affiliations:** 1 Applied Economics Department, Universitat de València, Av. Tarongers s/n., 46022 Valencia, Spain; 2 Research Centre for Health Economics and Management, Universitat Politècnica de València, Camino de Vera S/N., 46022 Valencia, Spain

**Keywords:** Public-private partnership, Hospital efficiency, Health management, Data envelopment analysis

## Abstract

Public-private partnership (PPP) initiatives are extending around the world, especially in Europe, as an innovation to traditional public health systems, with the intention of making them more efficient.

There is a varied range of PPP models with different degrees of responsibility from simple public sector contracts with the private, up to the complete privatisation of the service. As such, we may say the involvement of the private sector embraces the development, financing and provision of public infrastructures and delivery services.

In this paper, one of the oldest PPP initiatives developed in Spain and transferred to other European and Latin American countries is evaluated for first time: the integrated healthcare delivery Alzira model.

Through a comparison of public and PPP hospital performance, cost and quality indicators, the efficiency of the PPP experience in five hospitals is evaluated to identify the influence of private management in the results.

Regarding the performance and efficiency analysis, it is seen that the PPP group obtains good results, above the average, but not always better than those directly managed. It is necessary to conduct studies with a greater number of PPP hospitals to obtain conclusive results.

**Electronic supplementary material:**

The online version of this article (doi:10.1186/s13561-016-0095-5) contains supplementary material, which is available to authorized users.

## Background

In the last decade there has been a proliferation in different modalities of public-private partnership (PPP) for public service provision such as health, both in developed [1, 2] and developing countries [3, 4]. This provision of public infrastructure or services by the private sector bears significant risk and management responsibility. The private responsibility varies from full privatization to a mere management contract. Different models involve diverse forms of risk management, incentive, financing and payment of structures [5].

PPP models within the public health system have boomed during the period of economic crisis. One of the reasons behind this choice is that private sector has a greater capacity to handle the investment in hospitals, thus freeing the public sector from the considerable initial investment [6]. In defense of the PPP model, the argument has also been used that the public sector also has greater incentives for construction investment leading to operational cost reduction [7].

In the Spanish context especially, the variety of health management models is favored by the administrative structure and the division of political responsibilities. In Spain, health care beneficiaries and standards were defined centrally, but since 2002, when the decentralization process for health care responsibilities concluded, the responsibility for services delivery and funding has been devolved to the 17 Autonomous Communities (AC). Act 15/1997 provided for new forms of health system management, including contracting administrative concessions [8]. The health financing for each Spanish region is part of the mainstream regional financing system [9, 10].

The first Public Private Partnership (PPP) initiative in Spain is known as the Alzira model, after the town in the south-east of the European Mediterranean where the first hospital was built under this modality. This model consists of a private contractor building and operating the hospital, with a contract to provide care for a defined population. The adjudication of the contract to manage (administrative concession) is carried out by public tender. Following this framework, in 1999 a consortium consisting of an insurance company, banks and construction companies (*Rivera Salud SA)*, began operations in Alzira with the Hospital de la Ribera. Initial financing problems of sustainability were solved in 2002 with a refinancing deal [11].

In the Valencian Community (VC) Region (4.7 million inhabitants) there are 24 health districts, which are further divided into health zones with a primary health center. Each district has a hospital for specialist care, inpatient and outpatient. Therefore 20 % of VC population is covered by a PPP contract, with 5 health districts currently following this model: Alzira (since 1999), Torrevieja (since 2006), Denia (since 2009), Manises (since 2009) and Vinalopo (since 2010).

The objective of this type of contract is the management of integrated health care and is financed as follows: a premium per capita (of population covered with designated doctor) is assigned, and inter-center movements (from population of other departments) and the population from other Autonomous Communities are also invoiced. The activity of the concession is supervised by a commissioner from the Regional Health Department (*Conselleria*) [12].

In spite of the fact that PPP has generated interest in the academic world, the existing literature to date is still fragmented and focused on the cost aspect of management mainly in the Private Finance Initiative (PFI) [1, 5, 13, 14]. However, especially in the health sector, a holistic analysis of hospital performance is necessary, and not only a mere analysis of cost [12].

For Spain, there is little empirical, conclusive literature on the subject [15], which means opting for a PPP model may be considered to still be a political choice.

Even though the Alzira model has been the most widespread PPP model over the last decade, not only in the VC but also in other AC such as Madrid, there is still no empirical analysis which allows us to definitely back this model of hospital management.

Nevertheless, the coexistence of both models (PPP and public hospitals) in the same territory demands a deep analysis to adopt rational criteria in taking decisions about the suitability of each model. To achieve this objective it is fundamental that homogeneous and adequate information is available in order to be able to undertake empirical analyses of the performance of the PPP model in the long term.

Therefore, given the lack of up to date empirical analysis in this field, we consider our paper highly suitable in order to provide some evidence supported by a robust data base.

With these premises, the goal of this work is to empirically analyze the available data in order to establish a comparative analysis between public hospitals and the PPP model, with the purpose of identifying possible strong and weak points in both models and establishing bases for rational decisions on the appropriateness of each model.

The structure of this paper is the following: Section 2 is devoted to explaining in detail the data analyzed, that is, describing data sources and the main variables of the study. In addition, we define the methodology implemented. Section 3 details results provided by the empirical analysis. In section 4 we discuss findings and limitations of the research. Finally, section 5 synthetically describes the conclusions.

## Methods

### Data source

We used information on cost, production, hospital capacity, and quality indicators from 24 Valencian hospitals for the 2009–2010 periods. Five of the hospitals studied are PPP and nineteen are public. For some analyses shown in the following section, reliable information was only available for 3 of the 5 PPP operated hospitals.

For the public hospitals the source of the economic information regarding costs is the System of Economic Information (SIE) of the Regional Health Department (*Conselleria de Sanitat)*. The data regarding the costs of the PPP hospitals was obtained through a fill-in form, as they are not included in the SIE information system; therefore, it was adapted to the SIE criteria in order to make it comparable.

The data regarding quality came from the Management Agreements between the Hospitals and Regional Health Department, a ranking based on 95 indicators per Health District. Performance data on structure and health care attention were provided by the Minimum Data Set for admissions (MDS) and the Information System for Health care Activity (ISHA). All these sources were gathered by the Regional Health Department.

The following is a short glossary of the main variables used in this work^1^:

- Case Mix Index (CMI) adjusted admission (CMI x admissions) as a homogeneous measure of the hospital production.
- Adjusted patients (AP). We standardized all patients by applying the equivalent standards commonly used in this field to each patient in order to obtain comparable patients. Table 1 shows the specific weights applied to the adjustments [16].^2^

**Table 1:** Adjusted patients weights

Surgical processes	DRGs^3^ weights
Hospital processes	DRGs weights
First outpatient visits	0.033
Follow up outpatient visits	0.02
Emergencies	0.04

Source: IASIST 2009 [16]
- Total Cost. This variable is composed of the sum of medical staff cost, non-medical staff cost, medical material cost and pharmacy cost.
- Model of management. This is a dichotomy variable in which public hospitals, denoted by an H, took a value 0, and PPP Hospitals denoted by a C, took a value 1.
- Management agreement score. This variable was introduced in Valencian hospitals from 2004. The regional government and health area managers set several goals regarding efficiency and quality indicators. The achievement of these goals determines the management agreement score.

For this research we had data made available separately by services. Specifically, we analyzed data from medical inpatient, surgery, emergency and outpatient services.

### Analysis and measures

Firstly, we used the most suitable t-statistic for each variable in order to individuate significant performance differences between PPP hospitals and public hospitals. Normality of variables was previously checked through a Kolmogorov-Smirnov test in order to apply the proper t-statistic for mean and median differences.

Secondly, we ran a linear regression analysis aimed to identify which variables had a greater effect on the cost variability for each service.

Finally, an efficiency analysis through the Data Envelopment Analysis (DEA) model was run to rank hospitals according their efficiency performance [17–19]. The DEA model provided a ranking of overall hospital performance. For running the DEA model the following variables were selected^4^:

- Input variables: Human Resources cost, other costs, number of beds and number of operating rooms.
- Output variables: adjusted patients (surgical area), overall score in the Management Agreements, adjusted admissions and adjusted outpatients.

## Results

**Fig. 1:**
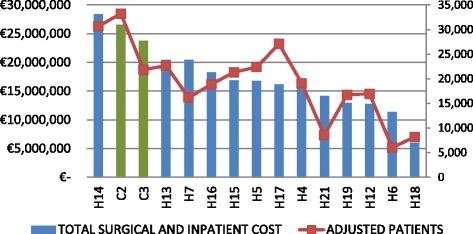
Cluster 1 (Medium and small hospitals)

**Fig. 2:**
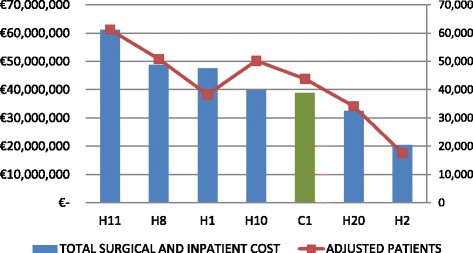
Cluster 2 (big hospitals)

In order to obtain a general overview of the relation between hospitals’ cost and production, a double variable graphic was designed. First, through a cluster analysis, we divided hospitals in two groups, medium/small hospitals and large hospitals. Clusters were set up by introducing structural and performance variables. Figures 1 and 2 show hospital production measured as adjusted admissions and cost for cluster 1 and 2 respectively, in order to summarize hospital performance. PPP hospitals are denoted by a C and public hospitals by an H.

For the large hospitals group, Fig. 2 shows a better performance in hospitals H1, H8, H10, C1 and H20, because, in relative terms, they produce more adjusted patients with less cost.

Adjusted patients were selected for designing the above graphics as it is the variable which best exemplifies hospital production. However, a t-statistic test was applied singly for each variable in order to individuate specific performance differences between public and PPP hospitals. After analyzing all available variables in the study, we only found significant differences in the performance between PPP and public hospitals in the set of variables shown in Table 2.

**Table 2:** Significant differences between management models

Better performance in PPP hospitals		
	PPP hospitals	Public hospitals
First consultations*	73050.80	48824.95
Wait for first consultations (days)*	14.52	20.06
Outpatient replacement rate*	80.67 %	60.09 %
MR equipment**	1	0
Management agreements score**	84.18	73.52
Rate of hip fracture operations with more than 2 days delay**	0.169	0.588
Better performance in public hospitals		
Medical material cost in emergency**	725782.24	248565.34

*T-statistic for average differences (*p* < 0.05)

**Mann-Whitney test for median differences (*p* < 0.05)

As Table 2 shows, statistically significant differences were found in seven variables. Public hospitals perform significantly better in “Emergency material cost”. For the others six variables, PPP hospitals show better results.

However, these results should be taken with caution, due to the sample of public hospitals being quite larger than the PPP sample.

Secondly, the results of the regression analysis show the most important variables to determine cost in each area. In Table 3, typified coefficients from the regression analysis are shown. In the medical inpatient area, “Number of beds” is the variable that has a higher effect on cost. “Model of management” also has an effect on cost but much weaker.

**Table 3:** Regression analysis results

	Medical area	Surgical area	Outpatient consultations	Emergency
	Typified coefficient			
Number of beds (medical inpatient area)	0.955			
Model of management	0.247			
Adjusted patients (surgical area)		0.598		
Number of beds (Surgical area)		0.419		
Examination rooms			0.888	
Adjusted patients (emergency area)				0.80
Plaster cast rooms (emergency area)				0.236
R^2^	0.860	0.981	0.776	0.883
*p* < 0.05	0.000	0.000	0.000	0.000

The sign of the non-standardized coefficient is positive; therefore, in this area PPP management increases costs. For the surgical area, variables that significantly affect cost are “Adjusted patients” and “Number of beds”. In the outpatient area, we individuate only “Examination rooms” as the variable that significantly affects cost. Finally, in the emergency area “Adjusted patients” is the variable that most affects cost. Also “Plaster cast rooms” has a significant effect, but very weak.

Finally, an efficiency analysis through the DEA model was run, in order to obtain a general overview of hospitals’ performance.

**Fig. 3:**
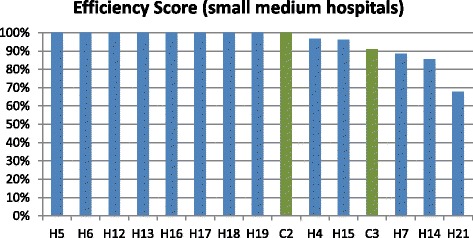
Efficiency analysis for small and medium hospitals (cluster 1)

**Fig. 4:**
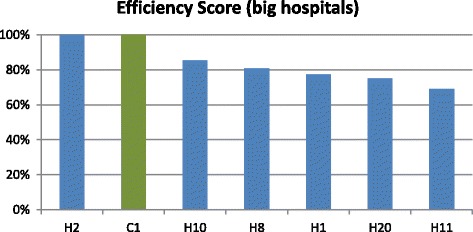
Efficiency analysis for large hospitals (cluster 2)

In addition, through a previous cluster analysis, we divided hospitals in two groups. In this way, we can compare hospitals with somehow homogeneous structural characteristics. In this analysis, only three PPP hospitals were included due to missing data in some of the selected input and output variables. Figures 3 and 4 show results of the efficiency analysis.

As shown in Fig. 3, within cluster 1 DEA analysis classified just one of the two PPP hospitals of the group as efficient. In cluster 2 (Fig. 4), only two hospitals were classified as efficient, one being a public hospital and the other a PPP hospital.

## Discussion

This paper analyzed one of the public-private partnership models within the health services field, specifically focusing on the Alzira model, which is the most widespread PPP model in Spain. We studied the case in which a private company delivers both the hospital service and the primary care for a designated geographic area, so public-private partnership involves full-service provision [11].

As our results show, the Alzira model presents some strengths in comparison to the public hospitals analyzed, giving better performance in some of the studied variables. We found that PPP improves some quality indicators, such as reduced delays in waiting lists. Even if this result agrees with literature [6], it should be taken with caution due to the limited PPP sample size.

In addition, the literature notes some possible advantages of the PPP model, as it releases the public sector from the substantial initial investment that construction of a hospital implies [20]. Furthermore, private management implies that the private companies should assume the financial risk instead of the public sector [6]. Additionally, some scholars confer a greater propensity to cost reduction and efficient performance in the private sector [1].

Nevertheless, we can also find some areas of less clarity in this kind of public-private collaboration. In the specific case of our study, the results are not conclusive enough to clearly acclaim the PPP model, due to our finding strengths and weaknesses in both the public and PPP models. To date, papers aimed at enhancing knowledge of PPPs in the health care sector have not reached forceful conclusions [13]

Furthermore, according to the literature, other considerations should be kept in mind regarding the PPP model. In the case of *Ribera Salud* (the company that manages PPP hospitals in VC) the assumption of the risk of the initial investment is not clearly delimited. Even if the operating risk was transferred to the health care providers, Ribera UTE is indirectly controlled by the VC and the original financing was provided by regional savings banks through a parent sponsor, therefore much of the risk falls back on the public partner [20]. This situation has recently changed since the equity of *Ribera Salud* includes an American Public Company (Centene Corporation).

Regarding the question of competition, we observe in our case study that there is a lack of real competition in assigning the concession contract. The magnitude of the initial investments means that the number of companies that can really bid for the concession tender are minimal. Therefore there is no really open competition [5]. There may also be an adverse risk selection in this model that is not sufficiently studied; the cost reducing orientation could promote incentives against complexity and cost against quality, as quality is not contractible and is hard to observe [6].

### Limitations

This paper is an important first step in the quantitative evaluation of PPP hospital performance in Spain. For future research some improvements need to be made. Firstly, a more balanced sample between the number of PPP and public hospitals analyzed would provide more conclusive results. Additionally, as only one of the five PPPs has been established and functioning for more than ten years, it is the only hospital providing extensive data, the others being open less than a year.

Secondly, it would be desirable to bring all economic information, from both PPP and public hospitals to a common database managed by the Regional Health Department, in order to have homogeneous information available for independent analysis of hospital performance. In this regard, Spain is one of the few European countries which do not have a central PPP agency aimed at applying a public sector comparator and standardized information [21].

Availability of accurate information about hospital activity is the key to permitting an empirical analysis of performance, which can establish rational decision-making criteria regarding the more suitable management model. In fact, lack of public access to data is one of the main problems which the majority of current research in this field has to face [20]. However, we achieved a complete database with financial, structural and activity information, both for public and PPP hospitals. We would thus like to emphasize that one of the main novelties and contributions of this paper is to run an empirical analysis resulting from a robust data base. Therefore, we conducted an unbiased analysis based merely on data analysis and literature review.

In this article, the cost of using private finance to build hospitals is not analyzed, as other authors propose [22], regarding additional cost of private over public finance found in the case of PFI Hospital in UK.

Beyond ideological criteria, independent and rigorous analysis with transparent data must be carried out to determine the effectiveness of these management models.

## Conclusion

In summary, regarding the performance and efficiency analysis, it is seen that the PPP group obtains good results, above the average for those directly managed, but not better in every case. Therefore, the results are not conclusive enough to clearly opt for one model of management; in both cases strengths and weaknesses were identified.

Nevertheless, our robust data base allowed us to begin taking the measure of Public-Private Partnership Hospitals in VC.

## Additional file

Additional file 1: Data base summary. (DOCX 31 kb)

## Abbreviations

AC: autonomous communities
AP: adjusted patients
CMI: case mix index
DEA: data envelopment analysis
DRG: diagnostics-related groups
ISHA: Information System for Health Care Activity
MDS: Minimum Data Set for Admissions
PFI: Private Finance Initiative
PPP: Public-Private Partnership
SIE: System of Economic Information
VC: Valencian Community

## Footnotes

1 The table with all variables used in the study can be consulted in Additional file 1: Appendix A.

2 Standard weights shown in the Table 1 were already reported in a previous research by IASIST 2009.

3 DRG: Diagnostic-related groups.

4 The selection of the variables was supervised and decided with a hospital manager. Variables selected are those most significant for the hospital performance.
