# Erratum to: ‘A cost and performance comparison of Public Private Partnership and public hospitals in Spain’

**DOI:** 10.1186/s13561-016-0098-2

**Published:** 2016-06-02

**Authors:** Maria Caballer-Tarazona, David Vivas-Consuelo

**Affiliations:** 1 Applied Economics Department, Universitat de València, Valencia, Spain; 2 Research Centre for Health Economics and Management, Universitat Politècnica de València, Valencia, Spain

Unfortunately, the original version of this article [1] contained an error. The author list was included incorrectly. Antonio Clemente-Collado was included as one of the authors by mistake. This author has now been removed from the author list as there was a conflict of interest. There will also be an update to correct this.
