# Supplementary material for: A cost and performance comparison of Public Private Partnership and public hospitals in Spain
Source: Health Econ Rev. 2016 May 14;6:17. doi: 10.1186/s13561-016-0095-5 (PMC4870542; doi:10.1186/s13561-016-0095-5)
Supplement: Additional file 1: — Data base summary. (DOCX 31 kb) [file 13561_2016_95_MOESM1_ESM.docx]

**Additional file 1: Appendix A**

| **HOSPITAL** | **PPP** | **CLUSTER** | **TOTAL COST** | **OUTPATIENT COST** | **EMERGENCY COST** | **SURGERY+INPATIENT COST** | **SURGERY COST** | **INPATIENT COST** | **HUMAN RESOURCES COST** |
| --- | --- | --- | --- | --- | --- | --- | --- | --- | --- |
| H1 | 0 | 2 | 71.280.962,69 | 12.210.327,55 | 11.482.893,23 | 47.587.741,91 | 20.180.951,31 | 27.406.790,61 | 57.232.038,60 |
| H2 | 0 | 2 | 33.855.291,82 | 8.152.432,17 | 5.254.950,55 | 20.447.909,10 | 9.792.319,94 | 10.655.589,16 | 28.013.496,91 |
| H3 | 0 | 0 | 9.257.986,96 | 2.174.109,15 | 3.242.106,80 | 3.841.771,01 | 3.398.515,56 | 443.255,45 | 8.297.726,69 |
| H4 | 0 | 1 | 26.961.501,66 | 3.410.860,52 | 7.431.826,82 | 16.118.814,32 | 9.578.305,13 | 6.540.509,19 | 23.403.941,55 |
| C1 | 1 | 2 | 55.892.711,73 | 8.848.327,22 | 8.154.466,40 | 38.889.918,12 | 19.040.085,07 | 19.849.833,05 | 46.236.030,12 |
| H5 | 0 | 1 | 29.548.843,31 | 5.818.345,56 | 6.993.991,92 | 16.736.505,83 | 9.132.385,56 | 7.604.120,27 | 23.364.312,94 |
| H6 | 0 | 1 | 17.876.507,89 | 1.991.826,83 | 4.506.345,04 | 11.378.336,02 | 3.552.748,29 | 7.825.587,73 | 15.487.326,00 |
| H7 | 0 | 1 | 30.913.317,67 | 4.492.420,76 | 6.021.148,32 | 20.399.748,59 | 7.712.485,37 | 12.687.263,23 | 27.418.684,27 |
| H8 | 0 | 2 | 85.236.424,18 | 23.621.538,33 | 12.765.080,46 | 48.849.805,39 | 22.827.152,90 | 26.022.652,49 | 58.187.678,18 |
| H9 | 0 | 0 | 9.010.244,86 | 952.528,51 | 0,00 | 8.057.716,35 | 7.455.104,70 | 602.611,65 | 6.266.768,45 |
| H10 | 0 | 2 | 63.824.724,68 | 14.174.652,18 | 9.978.653,41 | 39.671.419,09 | 24.918.644,11 | 14.752.774,98 | 48.802.838,94 |
| H11 | 0 | 2 | 89.699.718,32 | 14.545.126,57 | 13.973.359,69 | 61.181.232,06 | 28.903.090,35 | 32.278.141,71 | 67.425.693,70 |
| H12 | 0 | 1 | 22.112.310,95 | 4.747.112,90 | 4.606.908,85 | 12.758.289,20 | 7.983.699,71 | 4.774.589,49 | 18.539.117,95 |
| H13 | 0 | 1 | 27.692.580,03 | 3.325.165,32 | 3.914.840,64 | 20.452.574,07 | 9.609.662,53 | 10.842.911,54 | 14.825.540,77 |
| H14 | 0 | 1 | 47.901.539,32 | 8.714.839,59 | 10.803.167,23 | 28.383.532,50 | 16.659.545,89 | 11.723.986,61 | 37.831.333,81 |
| H15 | 0 | 1 | 26.163.242,54 | 4.468.054,73 | 4.857.260,26 | 16.837.927,56 | 9.121.805,06 | 7.716.122,50 | 19.807.791,35 |
| H16 | 0 | 1 | 31.211.605,76 | 4.576.807,57 | 8.409.063,19 | 18.225.735,00 | 9.575.458,40 | 8.650.276,60 | 24.489.777,36 |
| H17 | 0 | 1 | 29.463.970,05 | 5.588.539,46 | 7.689.528,10 | 16.185.902,49 | 10.813.154,07 | 5.372.748,42 | 23.126.413,19 |
| H18 | 0 | 1 | 10.040.431,03 | 1.965.098,06 | 2.155.471,35 | 5.919.861,62 | 3.688.715,91 | 2.231.145,71 | 7.612.742,97 |
| H19 | 0 | 1 | 23.003.507,82 | 5.170.700,37 | 4.890.319,11 | 12.942.488,34 | 9.582.337,18 | 3.360.151,16 | 19.975.982,29 |
| H20 | 0 | 2 | 48.177.826,12 | 9.378.316,91 | 6.370.705,66 | 32.428.803,55 | 19.152.104,33 | 13.276.699,22 | 36.625.971,42 |
| H21 | 0 | 1 | 22.607.578,74 | 7.044.652,28 | 1.430.074,03 | 14.132.852,43 | 7.113.311,54 | 7.019.540,89 | 18.849.279,72 |
| C2 | 1 | 1 | 38.833.154,57 | 6.082.867,56 | 6.222.285,41 | 26.528.001,60 | 11.384.260,65 | 15.143.740,95 | 30.106.850,22 |
| C4 | 1 | 0 | 0,00 | 0,00 | 0,00 | 0,00 | 0,00 | 0,00 | 0,00 |
| C3 | 1 | 1 | 37.121.737,25 | 6.899.286,98 | 6.509.297,11 | 23.713.153,16 | 11.246.101,51 | 12.467.051,65 | 28.939.572,49 |
| C5 | 1 | 0 | 0,00 | 0,00 | 0,00 | 0,00 | 0,00 | 0,00 | 0,00 |
| H22 | 0 | 3 | 178.927.610,22 | 38.306.312,72 | 25.016.119,78 | 115.605.177,71 | 53.716.794,62 | 61.888.383,09 | 135.662.344,17 |

| H | OUTPAT CASEMIX | SURG CASEMIX | TOTAL CASEMIX | AP INPATIENTS SURGERY | AP INPAT | AP SURG | AP EMER | AP OUTPAT | AP TOTAL | AP COST | AP INPATIENT COST | AP SURG COST | AP OUTPAT COST | AP EMERG COST |
| --- | --- | --- | --- | --- | --- | --- | --- | --- | --- | --- | --- | --- | --- | --- |
| H1 | 1,21 | 1,63 | 1,80 | 38.247,90 | 20.041 | 18.207 | 5.840 | 5.891 | 49.980 | 1.426 | 1.368 | 1.108 | 2.073 | 1.966 |
| H2 | 1,16 | 1,34 | 1,89 | 17.615,20 | 10.039 | 7.576 | 2.793 | 6.709 | 27.117 | 1.248 | 1.061 | 1.293 | 1.215 | 1.882 |
| H3 | 1,31 | 1,09 | 2,02 | 4.287,75 | 1.831 | 2.457 | 1.142 | 1.915 | 7.344 | 1.261 | 242 | 1.383 | 1.136 | 2.840 |
| H4 | 1,18 | 1,59 | 1,71 | 19.088,46 | 9.110 | 9.978 | 2.431 | 2.400 | 23.919 | 1.127 | 718 | 960 | 1.421 | 3.058 |
| C1 | 1,21 | 1,79 | 1,93 | 43.809,63 | 16.864 | 26.946 | 4.144 | 8.098 | 56.052 | 997 | 1.177 | 707 | 1.093 | 1.968 |
| H5 | 1,40 | 1,43 | 1,83 | 22.364,29 | 13.112 | 9.252 | 2.717 | 3.954 | 29.036 | 1.018 | 580 | 987 | 1.471 | 2.574 |
| H6 | 1,20 | 1,41 | 2,02 | 6.031,93 | 3.073 | 2.959 | 1.062 | 2.337 | 9.431 | 1.895 | 2.546 | 1.201 | 852 | 4.242 |
| H7 | 1,16 | 1,34 | 1,63 | 16.179,71 | 8.075 | 8.104 | 2.691 | 2.338 | 21.208 | 1.458 | 1.571 | 952 | 1.922 | 2.238 |
| H8 | 1,28 | 2,19 | 2,18 | 50.803,87 | 20.540 | 30.264 | 6.439 | 7.886 | 65.129 | 1.309 | 1.267 | 754 | 2.995 | 1.982 |
| H9 | 0,72 | 1,00 | 1,07 | 6.273,34 | 1.056 | 5.217 | 0 | 872 | 7.145 | 1.261 | 571 | 1.429 | 1.092 | 0 |
| H10 | 1,32 | 1,89 | 2,11 | 50.236,52 | 18.300 | 31.937 | 5.900 | 8.753 | 64.890 | 984 | 806 | 780 | 1.619 | 1.691 |
| H11 | 1,21 | 2,20 | 1,97 | 61.231,95 | 26.264 | 34.968 | 5.990 | 6.628 | 73.850 | 1.215 | 1.229 | 827 | 2.194 | 2.333 |
| H12 | 1,32 | 1,62 | 1,84 | 16.942,76 | 10.541 | 6.402 | 1.956 | 3.023 | 21.922 | 1.009 | 453 | 1.247 | 1.570 | 2.356 |
| H13 | 1,16 | 1,49 | 1,60 | 22.671,90 | 14.142 | 8.530 | 2.373 | 3.493 | 28.539 | 970 | 767 | 1.127 | 952 | 1.649 |
| H14 | 1,25 | 1,80 | 1,86 | 30.643,41 | 16.972 | 13.671 | 4.159 | 4.469 | 39.271 | 1.220 | 691 | 1.219 | 1.950 | 2.598 |
| H15 | 1,10 | 1,34 | 1,51 | 21.335,01 | 12.515 | 8.820 | 2.773 | 2.942 | 27.050 | 967 | 617 | 1.034 | 1.519 | 1.751 |
| H16 | 1,25 | 1,64 | 1,74 | 18.859,75 | 11.307 | 7.553 | 2.593 | 2.350 | 23.803 | 1.311 | 765 | 1.268 | 1.947 | 3.243 |
| H17 | 1,23 | 1,58 | 1,74 | 27.047,07 | 14.868 | 12.180 | 3.433 | 4.092 | 34.573 | 852 | 361 | 888 | 1.366 | 2.240 |
| H18 | 1,18 | 1,44 | 1,87 | 8.177,58 | 4.443 | 3.735 | 1.383 | 2.318 | 11.878 | 845 | 502 | 988 | 848 | 1.559 |
| H19 | 1,08 | 1,57 | 1,68 | 16.733,62 | 9.016 | 7.717 | 2.464 | 3.040 | 22.237 | 1.034 | 373 | 1.242 | 1.701 | 1.985 |
| H20 | 1,28 | 1,91 | 1,97 | 34.148,47 | 16.780 | 17.369 | 3.819 | 5.634 | 43.602 | 1.105 | 791 | 1.103 | 1.664 | 1.668 |
| H21 | 1,46 | 1,29 | 2,01 | 8.529,10 | 3.756 | 4.773 | 531 | 3.570 | 12.630 | 1.790 | 1.869 | 1.490 | 1.974 | 2.691 |
| C2 | 1,35 | 1,78 | 1,92 | 33.234,59 | 15.457 | 17.778 | 2.676 | 5.041 | 40.952 | 948 | 980 | 640 | 1.207 | 2.325 |
| C4 | 1,39 | 1,65 | 2,03 | 24.519,32 | 11.784 | 12.736 | 2.944 | 5.332 | 32.795 | 0 | 0 | 0 | 0 | 0 |
| C3 | 1,03 | 1,47 | 1,61 | 21.845,67 | 9.109 | 12.737 | 2.171 | 4.160 | 28.177 | 1.317 | 1.369 | 883 | 1.658 | 2.998 |
| C5 | 1,16 | 1,39 | 1,72 | 11.546,74 | 5.329 | 6.218 | 1.857 | 2.271 | 15.675 | 0 | 0 | 0 | 0 | 0 |
| H22 | 1,31 | 2,65 | 2,31 | 94.721,82 | 35.764 | 58.958 | 8.193 | 11.288 | 114.203 | 1.567 | 1.730 | 911 | 3.394 | 3.053 |
